# Cystathionine beta synthase modulates senescence of human endothelial cells

**DOI:** 10.18632/aging.100491

**Published:** 2012-10-18

**Authors:** Eva Albertini, Rafał Kozieł, Angela Dürr, Michael Neuhaus, Pidder Jansen-Dürr

**Affiliations:** Institute for Biomedical Aging Research (IBA), University of Innsbruck, 6020 Innsbruck, Austria

**Keywords:** cystathionine beta synthase, senescence, molecular biology of aging, transsulfuration

## Abstract

Availability of methionine is known to modulate the rate of aging in model organisms, best illustrated by the observation that dietary methionine restriction extends the lifespan of rodents. However, the underlying mechanisms are incompletely understood. In eukaryotic cells, methionine can be converted to cysteine through the reverse transsulfuration pathway thereby modulating intracellular methionine availability. Whereas previous results obtained in yeast and fruit flies suggest that alterations in the reverse transsulfuration pathway modulate the rate of aging, it is not known whether this function is conserved in evolution. Here we show that depletion of cystathionine beta synthase (CBS), a rate limiting enzyme in the reverse transsulfuration pathway, induces premature senescence in human endothelial cells. We found that CBS depletion induces mild mitochondrial dysfunction and increases the sensitivity of endothelial cells to homocysteine, a known inducer of endothelial cell senescence and an established risk factor for vascular disease. Our finding that CBS deficiency induces endothelial cell senescence *in vitro*, involving both mitochondrial dysfunction and increased susceptibility of the cells to homocysteine, suggests a new mechanism linking CBS deficiency to vascular aging and disease.

## INTRODUCTION

Availability of methionine is known to modulate the rate of aging in model organisms, best illustrated by the observation that dietary methionine restriction extends the lifespan of rodents [1]. However, the underlying mechanisms are incompletely understood. In eukaryotic cells, methionine can be converted to cysteine through the reverse transsulfuration pathway, in particular under conditions where methionine levels are high [2]. When methionine levels drop, the trans-methylation pathway is activated instead, leading to methionine production [3]. It is conceivable that the activity of these two pathways, by modulating intracellular methionine availability, affects the rate of aging; however, this hypothesis remains to be proven. Two genes acting in the reverse transsulfuration pathway were identified as novel modulators of chronological lifespan in yeast *S. cerevisiae*. Thus, deletion of the *CYS4* gene, the yeast ortholog of human cystathionine beta synthase (CBS), resulted in a significant lifespan extension. The conversion of homocysteine to cystathionine by CBS depends on pyridoxal 5'-phosphate (PLP), and lifespan extension was also observed in a *PDX3* deletion strain, deficient for the synthesis of PLP [4]. These data suggest that alterations in the transsulfuration pathway affect the aging phenotype in yeast. Similar conclusions have recently been reported for the role of the transsulfuration pathway in aging fruit flies. Thus, dietary protein restriction reduced levels of protein translation in Drosophila, largely caused by increased metabolic commitment of methionine cycle intermediates to transsulfuration. Endogenous dCBS activity was increased in extracts prepared from diet-restricted flies compared with extracts from fully fed animals. Of interest, overexpression of dCBS was sufficient to increase longevity [5]. Collectively, these findings strongly suggest that alterations in the reverse transsulfuration pathway modulate the rate of aging in lower eukaryotic model organisms. However, it is not known whether this function is conserved in evolution. In the present communication, we addressed the question if changes in CBS activity would affect aging in a model of human cellular senescence.

## RESULTS

### CBS depletion induces premature senescence in human endothelial cells

**Figure 1:**
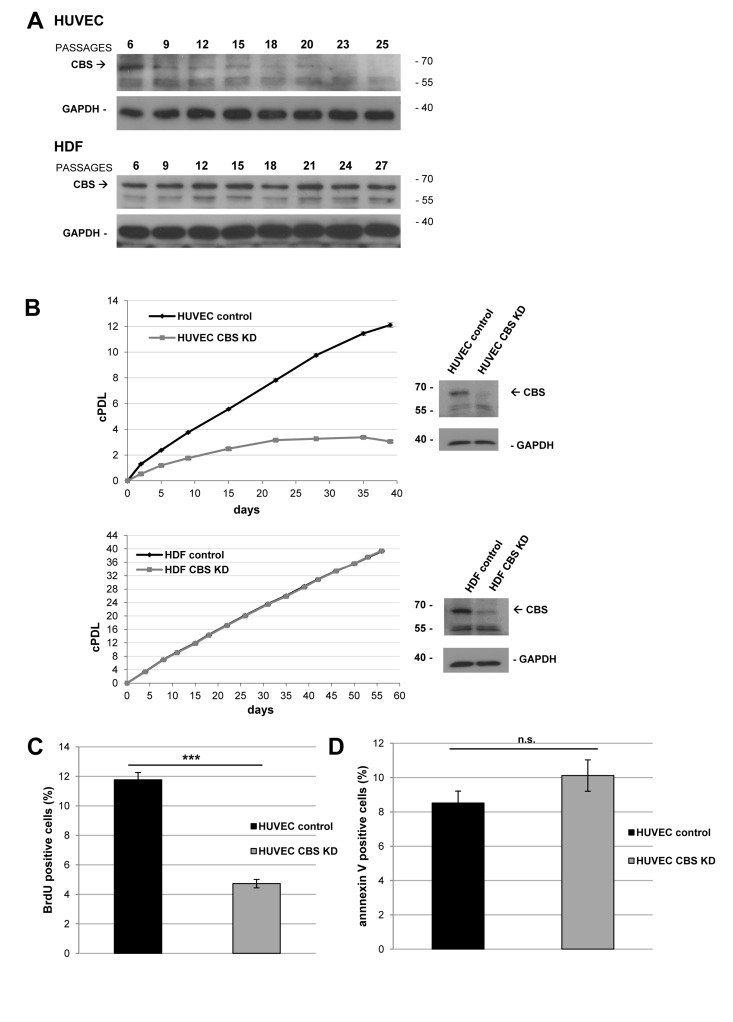
CBS depletion reduces the rate of cell proliferation (**A**) Protein was isolated from HUVEC and HDF in passage 6 (young) and subsequent passages as indicated; HUVEC reached senescence at passage 25, whereas HDF reached senescence at passage 27. CBS levels were determined by Western blot analysis, GAPDH served as loading control. (**B**) *Left panels*: CBS gene expression was inactivated by lentiviral shRNA vectors in early-passage HUVEC and HDF, as indicated. Cumulative population doublings (cPDL) were calculated, beginning 12 days after infection. Growth curves represent three independent experiments in each case. *Right panels*: CBS knockdown was verified by Western blot. (**C**) Bar diagram displaying relative percentage of BrdU positive (= proliferating) HUVEC 42 days after infection with lentiviral shRNA vectors. The percentage of BrdU positive cells was determined using flow cytometry; data are represented as mean ± SE (n=3). ***: p<0.001. (**D**) Bar diagram displaying relative percentage of annexin V positive (= apoptotic) HUVEC 42 days after infection with lentiviral shRNA vectors. The percentage of apoptotic cells was determined using flow cytometry; data are represented as mean ± SE (n=5). n.s: not significant

**Figure 2:**
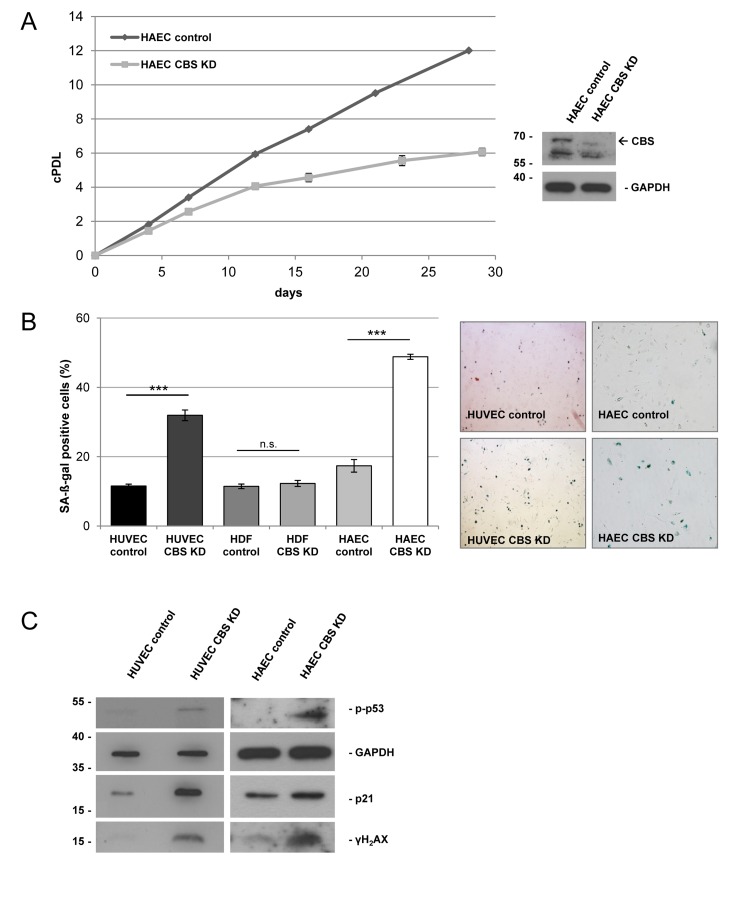
CBS depletion induces premature senescence in human endothelial cells CBS gene expression was inactivated by lentiviral shRNA vectors in early-passage HUVEC, HAEC and HDF, as indicated. (**A**) *Left panel*: Cumulative population doublings (cPDL) of CBS-depleted HAEC were calculated beginning 12 days after infection. Growth curve represents three independent experiments. *Right panel*: CBS knockdown was verified by Western blot. (**B**) The percentage of SA-ß-gal positive cells was determined 20 days after infection; data are represented as mean ± SE (n=3). ***: p<0.001, n.s: not significant. Micrographs show representative pictures of HUVEC and HAEC stained for SA-ß-gal. (**C**) Protein was isolated from control and CBS-depleted HUVEC and HAEC, as indicated. Protein levels were determined by Western blot, GAPDH served as loading control.

CBS expression decreased gradually with cellular aging in human umbilical vein endothelial cells (HUVEC), which are used as a model for vascular aging and endothelial dysfunction [6]. In contrast, CBS expression levels were not altered in senescent human dermal fibroblasts (HDF), suggesting a cell type specific effect (Fig. 1A). Depletion of CBS was obtained by lentiviral CBS-targeting shRNA (Fig. 1B), whereas infection with empty vector (Fig. 1B) or a non-targeting shRNA (data not shown) did not affect CBS levels. CBS depletion led to decreased cell numbers in HUVEC but not HDF (Fig. 1B); it also significantly reduced the rate of cell proliferation, measured by BrdU incorporation studies (Fig. 1C), but had no effect on the rate of apoptotic cell death (Fig. 1D). CBS knockdown also reduced the proliferative capacity of human aortic endothelial cells (HAEC) (Fig. 2A), included as an additional control. Both in HUVEC and HAEC, CBS knockdown led to a premature accumulation of cells staining positive for senescence associated ß-galactosidase (SA-ß-gal) (Fig. 2B), whereas the proportion of SA-ß-gal-positive cells was not altered by CBS depletion in HDF (Fig. 2B). Both in HUVEC and in HAEC, CBS depletion induced the expression of molecular senescence markers, such as p21^WAF-1^ and γ-H_2_AX (Fig. 2B), suggesting that CBS depletion induces premature senescence in human endothelial cells.

### CBS knockdown induces mild mitochondrial dysfunction

**Figure 3:**
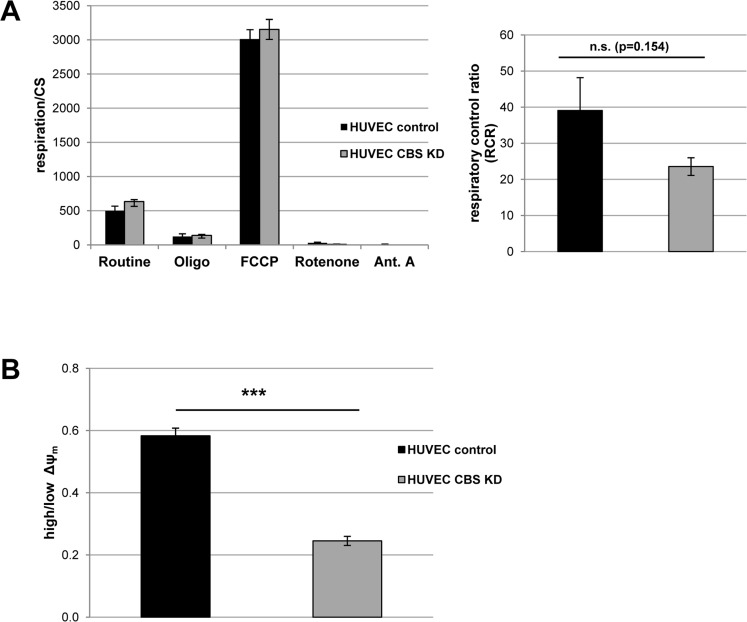
CBS depletion induces mild mitochondrial dysfunction (**A**) *Left panel:* CBS-depleted HUVEC were subjected to high resolution respirometry. The experimental regime started with routine respiration, followed by addition of oligomycin, and stepwise titration of FCCP. Finally, respiration was inhibited by sequential addition of rotenone and antimycin A (Ant.A). Respirometry results were normalized to citrate synthase activity; data are represented as mean ± SE (n=6 and 7 for CBS knockdown and control, respectively). *Right panel:* Respiratory control ratio was determined as the ratio of uncoupled respiration to oligomycin-inhibited respiration. (**B**) The electric potential of the inner mitochondrial membrane was measured *in situ* using flow cytometry in intact cells stained with the JC-1 fluorescent probe. The ratio of cells with high to cells with low mitochondrial membrane potential was calculated as described in the methods. Data are represented as mean ± SE (n=3).

From studies in model organisms, it is known that methionine restriction increases lifespan via alterations of mitochondrial function [1]. Since CBS knockdown can be considered as a genetic condition to increase methionine concentration, we addressed the question if CBS knockdown would affect mitochondrial function. Whereas depletion of CBS did not significantly influence oxygen consumption, the respiratory control ratio was reduced in CBS knockdown cells (Fig. 3A), although differences did not reach statistical significance. Moreover, mitochondrial membrane potential was significantly reduced in CBS depleted cells (Fig. 3B). Together these data suggest that depletion of CBS leads to mitochondrial membrane depolarization and partial uncoupling of respiration, conditions that were shown to induce senescence in human fibroblasts [7, 8].

### CBS overexpression extends lifespan of human endothelial cells

**Figure 4:**
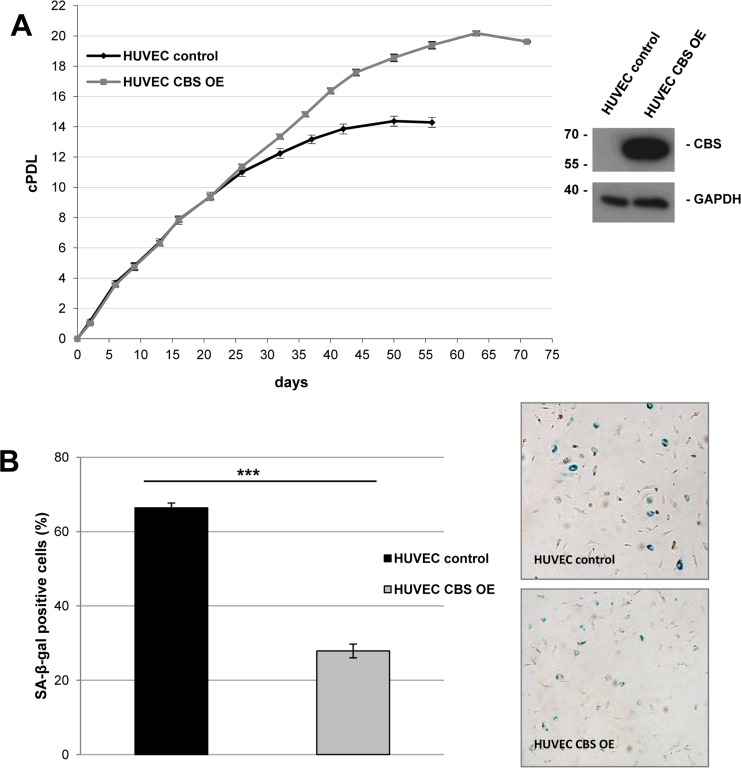
CBS overexpression delays premature senescence in human endothelial cells (**A**) *Left panel*: Cumulative population doublings of CBS overexpressing (OE) and control HUVEC were calculated, beginning 11 days after infection. Growth curves represent three independent experiments in each case. *Right panel*: CBS Western blot after lentiviral infection with the CBS overexpression and control construct. (**B**) *Left panel*: Bar diagram displaying relative percentage of SA-ß-gal positive cells 46 days after lentiviral infection with the CBS overexpression and control construct. Data are represented as mean ± SE (n=3). ***: p<0.001 *Right panel*: Representative micrographs of cells stained for SA-ß-gal activity.

**Figure 5:**
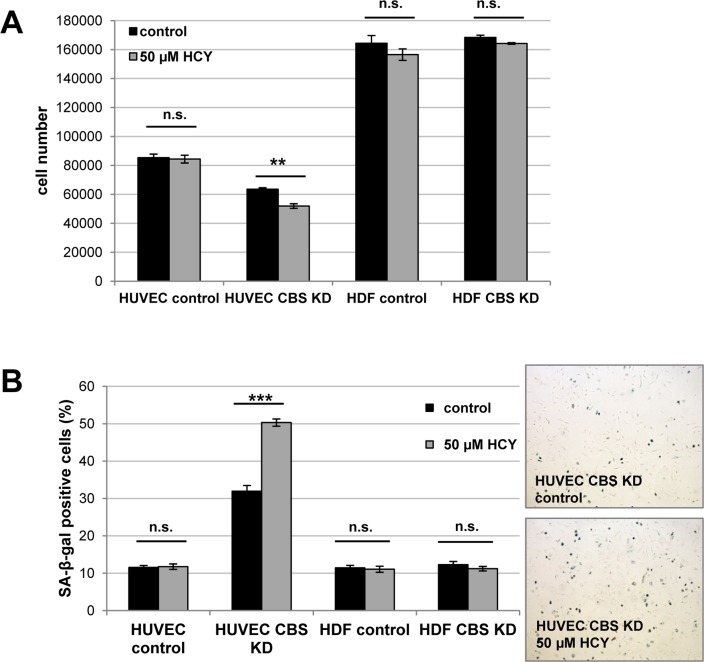
CBS depletion increases susceptibility of HUVEC to homocysteine-induced senescence CBS gene expression was inactivated by lentiviral shRNA vectors in early-passage HUVEC and HDF, as indicated, and homocysteine (Hcy) was added. Cells were counted after 72 h (*panel A*), and the percentage of SA-ß-gal positive cells was determined after 84 h (*panel B*). Data are represented as mean ± SE (n=3). Micrographs show representative pictures of cells stained for SA-ß-gal. ***: p<0.001, **: p<0.01, n.s.: not significant.

To address the question whether CBS directly controls entry of human endothelial cells in cellular senescence, the protein was expressed from a lentiviral vector in early passage HUVEC, which were then monitored over several passages. CBS overexpression induced a significant extension of HUVEC replicative lifespan (Fig. 4A) and postponed the appearance of senescence markers (Fig. 4B). Together, the findings reported here identified CBS as a rate-limiting enzyme controlling replicative senescence of human endothelial cells. We also addressed the possibility that the ability of CBS to delay cellular senescence may be linked to alterations in the level of homocysteine (Hcy). Since Hcy is the physiological substrate of CBS [9], depletion of CBS can be expected to increase the levels of Hcy. Whereas technical difficulties precluded the determination of intracellular Hcy concentration in our experimental system, we tested the response of control and CBS-depleted cells to the addition of extracellular Hcy, well known for its ability to induce premature senescence in human endothelial cells [10]. Addition of Hcy in various concentrations had no effect on proliferation and senescence in HDF (Fig. 5 and data not shown), whereas the addition of a very low dose of Hcy (50 μM; see ref. [10]) to CBS-depleted HUVEC but not control HUVEC led to a significant decrease in cell proliferation (Fig. 5A) and increased the percentage of SA-ß-gal positive cells (Fig. 5B). Together, these findings suggest that the ability of Hcy to induce premature senescence in endothelial cells is enhanced by CBS depletion.

## DISCUSSION

In the present communication, we observed that expression of cystathionine beta synthase (CBS) declined with serial passaging in human endothelial cells but not in other cell types. We also demonstrated that gene silencing of CBS by shRNA induced premature senescence, whereas senescence could be postponed by CBS overexpression in normal human endothelial cells. We also addressed potential mechanisms by which CBS affects the senescence phenotype. On the one hand, CBS knockdown induced mild respiratory uncoupling and reduced mitochondrial membrane potential. On the other hand, CBS knockdown increased the vulnerability of human endothelial cells to homocysteine, a well-known inducer of cellular senescence, which was described by others as risk factor predisposing to endothelial dysfunction and disease [11-13].

It is known from previous studies that signaling through mammalian target of rapamycin (mTOR), a known sensor of amino acid availability, can influence the senescent phenotype by at least two distinct pathways. On the one hand, it is known that mTOR signaling contributes to the senescent phenotype and thereby drives a shift from quiescence to senescence [14], probably via increased signaling through TOR Complex 1 [15]. On the other hand, mTOR, by driving proliferation, counteracts cell cycle arrest, potentially masking its effects on replicative lifespan [16]. Preliminary data suggest that CBS knockdown, while inducing cellular senescence, reduces mTOR activity in human endothelial cells (E. Albertini, unpublished), probably reflecting reduced availability of cysteine in such cells. However, more work will be required to elucidate the role of mTOR signaling in senescence induced by CBS downregulation. Whereas hypoxia inhibited the mTOR pathway and suppressed senescence in fibroblasts and retinal cells [17], the effect of hypoxia on senescence of human endothelial cells is currently unknown but warrants further investigation.

Previous data reported by us [4] and by others [5] have suggested that the transsulfuration pathway impacts on the rate of aging, probably by regulating intracellular availability of methionine. Importantly, deletion of CBS in yeast was shown to extend chronological lifespan [4], whereas in fruit flies, lifespan extension required CBS overexpression instead [5]. The role of CBS in lifespan regulation in mammals is currently unknown, although experiments with CBS^−/−^ mice clearly established that homozygous deletion of the CBS gene results in a severe disease phenotype with drastically shortened lifespan and healthspan [18]. In patients, CBS deficiency results in the accumulation of homocysteine in the serum [11, 13]. Severe homocysteinemia was also observed in mouse models for CBS deficiency; whereas CBS^−/−^ mice die a few weeks after birth [18], CBS ^−/+^ mice were established as a model for CBS deficiency. Increased serum levels of homocysteine induce damage to endothelial cells in CBS ^−/+^ mice [19] and in human patients with homocysteinemia [11]. Accordingly, elevated plasma total homocysteine is a risk factor for vascular disease [11], and premature arteriosclerosis [9]. It has been hypothesized that senescence of endothelial cells in the vessel wall contributes to vascular aging and disease [6]. In this communication, we show that CBS deficiency induces endothelial cell senescence *in vitro*, involving both mitochondrial dysfunction and increased vulnerability of the cells to exogenous homocysteine, suggesting a new mechanism linking CBS deficiency to vascular aging and disease.

## METHODS

### Cell culture

Human umbilical vein endothelial cells (HUVEC) were isolated and maintained according to the methods previously described [20]. Human aortic endothelial cells (HAEC) were purchased from Patricell. HUVEC and HAEC were propagated in endothelial cell growth medium (EBM CC-3121 supplemented with CC-4133, Lonza). Human diploid foreskin fibroblasts (HDF) were purchased from ATCC (Manassas, VA) (HFF-2 #SCRC-1042). HDF were propagated in Dulbecco's modified Eagle's medium (D5546, Sigma) supplemented with 10% heat-inactivated fetal bovine serum (Biochrom AG), 4 mM L-glutamine (Gibco) and 1% penicillin/streptomycin (Gibco). All cells were grown in an atmosphere of 5% CO_2_ at 37°C and were cultured in the same way as previously described [7].

### Lentiviral knockdown and overexpression of CBS

For the overexpression of CBS the lentiviral pLenti6/V5-DEST Gateway vector (Invitrogen) was used. Cloning included the TOPO cloning of CBS into pENTR/D-TOPO. This vector was used to introduce the CBS coding sequence into pLenti6/V5-DEST by recombination to generate the transfer vector pLenti6-CBS (for further details see Invitrogen's ViraPower Lentiviral Expression System manual). pLenti/V5-DEST was used as a negative control for overexpression. As a transfer vector for knockdown of CBS, we used the lentiviral pLKO.1-TRC short-hairpin vector from Addgene/Open Biosystems (United Kingdom). The following sequence was chosen: pLKO-61 (5'-CCG TCA GAC CAA GTT GGC AAA-3'). As a control for knockdown analysis the empty pLKO.1-TCR vector, as well as a control vector expressing a non-targeting shRNA (MISSION® Non-Mammalian shRNA Control Transduction Particles, Sigma-Aldrich) were used. Production of lentiviruses using 293FT cells and titering of the virus stocks was performed as described earlier [21]. For transfection 4×10^4^ early passage HDF and HUVEC were seeded to 6-well plates the day before. The medium was aspirated and replaced with 1 ml medium containing 8 μg/ml polybrene and CBS knockdown, overexpression or control lentiviruses, respectively (multiplicity of infection =2). The next day, medium was changed and the day after selection was started. For selection of CBS overexpressing and corresponding control cells 10 μg/ml blasticidin, for CBS knockdown and corresponding control cells 500 ng/ml puromycin was used. Beginning 10–14 days after transfection, the cumulative population doublings (cPDL) were calculated using the following equation: cPDL = (log(A) − log(B))/log2 (A: number of cells at the end of one passage; B: number of cells that were seeded at the beginning of one passage).

### Protein isolation

For the preparation of whole cell lysates, cells were washed twice with cold PBS and scraped off on ice in lysis buffer (50 mM Tris-HCl, 150 mM NaCl, 1% NP-40, 0.25% Na-deoxycholate, 1 mM EDTA, 100 nM Na3VO4, 1 mM NaF, 10 mM ß-glycerophosphate, pH 7.4) from a 10 cm dish with a rubber policeman. Cells were three times deep-frozen in liquid nitrogen and thawed and further kept on ice for 30 minutes. After centrifugation at 20,000 × g for 10 minutes at 4°C, supernatant was used to determine the protein concentration by DC Protein Assay Kit (Biorad, Austria).

### Immunoblotting

Equal amounts of protein were subjected to SDS gel electrophoresis (10–12.5% SDS/polyacrylamide gel) and transferred to PVDF membrane by wet electro-blotting (300 mA, 1 h) using the standard Western blot protocol. Immune-reactive proteins were detected using an enhanced chemiluminescence system (ECL+, Amersham Life Science, Germany). The following antibodies were used: mouse monoclonal anti-p21 (Pharmingen BD Biosciences), rabbit polyclonal anti-GAPDH (Santa Cruz), rabbit polyclonal anti-phospho-p53 (Ser15; Cell Signaling), rabbit polyclonal anti-H2A.X (Ser139; Cell Signaling) and mouse polyclonal anti-CBS (Abcam). As secondary antibodies polyclonal antibodies from Dako were used.

### Senescence-associated ß-galactosidase (SA-ß-gal) staining

The senescent status of the cells was verified by *in situ* staining for SA-ß-galactosidase as described before [22]. Briefly, cells that were grown on 6-well plates were washed three times with PBS and fixed with 2% formaldehyde/0.4% glutaraldehyde in PBS for 5 minutes at room temperature. Then they were washed again and incubated with ß-galactosidase staining solution (150 mM NaCl, 2 mM MgCl, 5 mM potassium ferricyanide, 5 mM potassium ferrocyanide, 40 mM citric acid, 12 mM sodium phosphate, pH 6.0, adding 1 mg ml-1 5-bromo-4-chloro-3-indolyl-ß-D-galactoside [X-gal] directly before use) and incubated for 24 h at 37°C without light exposure. The reaction was stopped by washing off the staining solution with PBS. Cells were covered with PBS and blue staining indicating the presence of SA-ß-gal can be detected under the microscope. To calculate the percentage of SA-ß-gal positive cells, stained cells were counted and related to the total cell number.

### Cell proliferation and apoptosis assays

Cell proliferation was assayed using the 5-bromo-2'-deoxy-uridine (BrdU) labeling and detection kit I (Roche, Vienna, Austria). BrdU incorporation was measured in 1 × 10^4^ cells using flow cytometry (FACS Canto II, Becton Dickinson, Heidelberg, Germany).

For detection of apoptosis, the cells were detached, washed once with PBS and incubated with 100 μl AnnexinV buffer containing 10 mM HEPES, 140 mM NaCl and 2.5 mM CaCl_2_ (pH 7.4) and 3 μl of AnnexinV-fluoresceinisothiocyanate (Pharmingen BD Biosciences, Heidelberg, Germany) for 15 minutes at room temperature. After the incubation, 300 μl of AnnexinV buffer were added to the cell suspension and 1 × 10^4^ cells were measured using a flow cytometer (FACS Canto II, Becton Dickinson).

### Determination of mitochondrial membrane potential

The electric potential of the inner mitochondrial membrane was measured *in situ* using flow cytometry in cells stained with the JC-1 (Molecular Probes) fluorescent probe [23]. 1 × 10^5^ cells were suspended in 2 ml of the culture medium supplemented with 0.5 μg ml-1 of JC-1. The cells were incubated for 30 minutes at 37°C, washed twice with PBS, and resuspended in PBS. JC-1 fluorescence was measured using flow cytometry (FACS Canto II, Becton Dickinson) in 1 × 10^4^ cells. In healthy cells, JC-1 enters the negatively charged mitochondria where it aggregates and fluoresces red. When mitochondrial potential drops, JC-1 exists as monomers in cytoplasm and fluoresces green. It allows to define two populations of cells, with high Δψ_m_ (high red, low green fluorescence) and low Δψ_m_ (low red, high green flourescence). Preincubation of the cells with mitochondrial uncoupler allowed defining the cell population with lowered Δψ_m_ (mean value of green channel fluorescence above 10^4^ (arbitrary units) and mean value of red channel fluorescence below 10^4^). All cells with lower green and higher red fluorescence were counted as cells with high Δψ_m_ (percentage of population).

### High-resolution respirometry

Cells were harvested, spun at 155 × g for 5 minutes, and finally resuspended in EGM. Cell number was determined using CASY 1 Cell Counter and Analyser System (Schärfe System, Reutlingen, Germany). Approximately 1 × 10^6^ intact cells were resuspended in 3 ml of EGM and applied for high-resolution respirometry using Oxygraph-2k (Oroboros Instruments, Innsbruck, Austria).

The experimental regime started with routine respiration (defined as respiration in culture medium without any additional substrates or effectors). After observing steady-state respiratory flux the ATP synthase inhibitor oligomycin was added, followed by uncoupling of OXPHOS by stepwise titration of carbonyl cyanide p-trifluoromethoxyphenylhydrazone (FCCP) up to optimum concentrations in the range of 2.5–4 μM. Finally, respiration was inhibited by complex I and complex III inhibitors rotenone (0.5 μM) and antimycin A (2.5 μM), respectively. DataLab software (Oroboros Instruments, Innsbruck, Austria) was used for data acquisition and analysis. The respirometry data were normalized to the mitochondrial mass marker enzyme citrate synthase activity as described [24]. Briefly, two portions of 300 μl of the sample were taken from the cell suspension stirred in the Oxygraph chamber before the chamber was closed for recording respiration. Samples were frozen in liquid nitrogen and stored at −80°C. Total cell lysate (100 μl) was added to 900 μl of medium containing 0.1 mM 5,5-dithio-bis-(2-nitrobenzoic) acid (DTNB), 0.5 mM oxaloacetate, 50 μM EDTA, 0.31 nM acetyl-CoA, 5 mM triethanolamine hydrochloride, and 0.1 M Tris/HCl (pH 8.1). The activity of citrate synthase was measured spectrophotometrically at 412 nm and 30°C.

### Homocysteine treatment

4×10^4^ cells were seeded on 6-well plates and grown in culture medium containing 50 μM homocysteine. Also, 4 μM copper(II) sulfate (CuSO_4_) was added to the culture medium, since previous studies have indicated that many biological effects of homocysteine require the addition of copper [10, 25]. Media was changed every 24 hours, and cells were harvested after 72 hours homocysteine treatment. Cell number was determined using CASY® Cell Counter and Analyser System (Schärfe System, Reutlingen, Germany). For SA-ß-gal analysis cells were seeded again on 6-well plates after counting in normal culture medium. Staining for SA-ß-gal was performed 12 hours after re-seeding.
